# Sensor Faults Isolation in Networked Control Systems: Application to Mobile Robot Platoons

**DOI:** 10.3390/s21206702

**Published:** 2021-10-09

**Authors:** Wijaya Kurniawan, Lorinc Marton

**Affiliations:** 1 Department of Electrical Engineering and Information Systems, University of Pannonia, 8200 Veszprem, Hungary; 2 Department of Electrical Engineering, Sapientia Hungarian University of Transylvania, 547367 Corunca, Romania; martonl@ms.sapientia.ro

**Keywords:** Unknown Input Observer, mobile robot platoons, weak fault isolation, sensor faults, networked systems

## Abstract

In networked control systems, sensor faults in a subsystem have a major influence on the entire network as the fault effect reaches the other subsystems through the network interconnections. In this paper, a fault diagnosis-oriented model is proposed for linear networked control systems that can be applied to the robotics platoon. In addition, this model can also be used to design distributed Unknown Input Observers (UIO) in each subsystem to accomplish weak sensor faults isolation by treating the network disturbances and fault propagation through the network as unknown inputs. A case study was developed in which the subsystems were represented by robots that are connected in a wireless communication-based leader-follower scheme. The simulation results show that the model successfully reproduces the expected behaviour of the robotics platoon in the presence of sensor faults. Furthermore, weak sensor faults isolation is also achieved by observing the residual signals produced by the UIOs in each of the subsystems.

## 1. Introduction

The theory of cooperative robotics deals with such groups of robots that communicate with each other to achieve some common goals. Such systems have always been an important topic to be researched because they can be applied to perform challenging tasks such as space exploration, domestic help, healthcare, military operations, and mobile Wireless Sensor Networks (WSN) [1]. In this era of Industry 4.0, robot platoons becomes more popular and have an increasing demand in the modern industrial environment because they can solve tasks that can not be done by only one robot [2]. Multiple robots can cooperate in accomplishing predefined tasks, such as goods transportation in the warehouses or monitoring and surveillance of production systems. However, there are many safety-critical issues to be handled here in the area of communication and control of these systems [3,4] for example the fault isolation problem for each robot in the platoons.

To achieve a common goal, besides local measurement from its attached sensors, each robot in the multi robotic systems also needs information from the others through a communication network. Because sensor faults on a robot can propagate through the network, fault isolation problems must be acknowledged so that these faults will not affect the others and disturb the coordination. There are many approaches to solve the fault diagnosis problem in control system [5]. A book by Varga et al. discusses fault detection and isolation from a computational perspective [6]. In the case of a model-based approach, a simple linear model is proposed to detect and isolate faults in electrical grids by Pozna et al. [7]. Gertler et al. proposed the design of dynamic parity relations for fault detection and isolation [8]. The optimization-based approach usually using either $H_{2}$ or $H_{\infty }$ has also been done by Song et al. and Niemann et al. [9,10]. Menke et al. and White et al. used the Kalman filter for fault estimation in aerospace systems [11,12]. There is also a book by Simani et al. explaining how to undertake fault diagnosis based on system identification techniques [13]. The current emerging trend in Artificial Intelligence (AI) has also been exploited by Michail et al, Nasiri et al, and Xiao et al. to undertake fault detection and isolation [14,15,16]. In a recent article, Li et al. have also made a summary about many methods which have been researched to solve this fault detection and identification problems [17].

Other than those already mentioned, Unknown Input Observer (UIO) is a popular approach in a model-based fault diagnosis because it can decouple the influence of unknown inputs on state estimation. In [18], it was shown that weak faults isolation can be achieved using this UIO. Weak faults isolation means that the faults in different sensors can be isolated when no simultaneous fault occurs. Residual generators are built from these UIOs for fault diagnosis purposes which are sensitive to some groups of faults but insensitive to others. Chakrabarty et al. have further explored the implementation of this UIO to accommodate bounded exogenous inputs and delayed measurements [19,20]. Xu et al. combined UIO with set-theoretic methods for robust fault detection purpose [21]. Sensor faults detection on a UAV using UIO has also been researched by Zuo et al. [22].

Because the network contributes to many unpredicted unknown inputs such as disturbance and fault propagation, this unknown inputs decoupling feature becomes more important in networked control systems. Thus, much research to implement UIO in this networked environment has been done. Taha et al. investigated state estimation conducted by UIO which is connected to an observed plant via network [23]. UIO for interconnected second-order system investigation has also been done by Shames et al. [24]. Chen et al. and Chakrabarty et al. explored the design of UIO for a class of interconnected non-linear systems [25,26].

In terms of fault diagnosis in a networked environment, Liu et al. and Shames et al. explored the utilization of UIO to detect a faulty agent in a multi-agent system [27,28]. Zhang et al. investigated how to do either actuator or sensor faults identification for each agent using a global UIO in networked control systems [29].

To take this research trend further, this paper proposes the design of a bank of local UIOs to detect and isolate faulty sensors in each subsystem of the robotics platoon, not just detecting faulty robots. The research questions we are going to answer are:

- How can we develop such sensor faults isolation method for each robot of the platoons using only conventional sensors (GPS, velocity sensor, radar) and network communication?
- How to compensate faults propagation effect in the communication network from one robot to the others so that it does not affect the sensor faults isolation process?
- How to implement the sensor faults isolation in a distributed way?

We do it by extending the UIO-based sensor faults isolation problem into networked control systems. This local faults isolation scheme has advantages in terms of scalability features compared to a global UIO. The concerned networked control systems for the implementation of these banks of UIOs are a controlled robots platoon inspired by Adaptive Cruise Control (ACC) system [30]. The proposed approach ensures the isolation of faults in sensors that are critical for safe platooning.

The new contributions of this paper are as follows:

1. We have developed a model for sensor faults diagnosis purposes for the robotics platoon.
2. We have proposed a general UIO-based distributed sensor faults isolation approach for a network of linear control systems.
3. Based on these two results, we have solved the weak sensor faults isolation problem in such robotics platoon that use conventional sensors: GPS-based localization; velocity sensor; and radar sensor.

The organization of this paper will be as follow: In Section 2, the existing Unknown Input Observer method for sensor faults isolation is recalled. In Section 3, sensor faults propagation and the proposed distributed sensor faults isolation in networked control systems using UIO are explained. In Section 4, the proposed modelling of a controlled robots platoon is discussed both in the fault-free case and faulty case. In Section 5, the implementation of weak sensor faults isolation on the robotics platoon is presented. In Section 6, the simulation results of sensor faults isolation in 5 robots moved together as a platoon in a leader-follower control scheme are discussed. Finally, conclusions and suggestions for future works are given in Section 7.

## 2. UIO-Based Sensor Faults Isolation

This section recalls general notions about UIO-based estimation and faults isolation, see [18] for details.

### 2.1. General Model of Unknown Input Observer (UIO)

Consider a fault free linear system injected with unknown inputs. In addition, also consider for the sake of convenience but without loss of generality, that the system’s outputs are not directly affected by the inputs. Hence, the fault free model can be written in the form of:

(1) $$\begin{array}{c} \begin{array}{cc} \dot{x}\left(t\right) & =Ax(t)+Bu(t)+Ed(t)\\ y(t) & =Cx(t) \end{array} \end{array}$$

where $x\left(t\right)\in R^{n}$ is the state vector, $y\left(t\right)\in R^{m}$ is the measured output vector, $u\left(t\right)\in R^{r}$ is the known control input vector, $d\left(t\right)\in R^{q}$ is the unknown inputs (or disturbance) vector. A, B, C, and E are known matrices with appropriate dimension.

An Unknown Input Observer (UIO) is a special observer, which despite of the presence of some unknown inputs d(t), it still produces a state estimation $\tilde{x}$ that converges to the real one if certain conditions hold. The state-space equation for this UIO’s state z(t) is:

(2) $$\begin{array}{c} \begin{array}{cc} \dot{z}\left(t\right) & =Fz(t)+TBu(t)+Ky(t)\\ \tilde{x}\left(t\right) & =z(t)+Hy(t) \end{array} \end{array}$$

where $\tilde{x}\left(t\right)\in R^{n}$ is the state estimation vector and $z\left(t\right)\in R^{n}$ is the UIO’s internal state vector.

By choosing the matrices *F*, *T*, *K*, and *H* to satisfy:

(3) $$\begin{array}{c} \begin{array}{cc} HCE & =E\\ T & =I-HC\\ F & =A-HCA-K_{1}C\\ K_{2} & =FH\\ K & =K_{1}+K_{2} \end{array} \end{array}$$

with *I* is the Identity Matrix, then $\lim_{t\to +\infty }\left(x-\tilde{x}\right)=0$.

The necessary conditions so that Equation (3) can be satisfied are:

(4) $$\begin{array}{c} \begin{array}{cc} & 1.\;rank(CE)=rank(E)\\ & 2.\;(C,A-HCA)\;is\;a\;detectable\;pair \end{array} \end{array}$$

The first condition is to ensure that *H* matrix in Equation (3) exists. It also indicates that the maximum size of the disturbances vector that can be decoupled can not exceed the number of independent measurements which are done. In other words, sensor faults isolation encourages redundancy either in the measurement or in the number of the used sensors. The second condition is a milder condition related to observability. If (C,A−HCA) is already observable, a pole placement method can be directly used to determine the value of $K_{1}$. If it is not observable, an observable canonical decomposition procedure must be performed to do a matrix transformation before using pole placement to find the value of $K_{1}$. The details of this procedure are explained in [31].

### 2.2. UIO for Sensor Faults Isolation

Sensor faults isolation refers to a process for determining in which sensor the fault has occurred. One way to accomplish this is by using structured residual signals. These structured residual signals are made in such a way that each of them is sensitive (or insensitive) to a certain group of faults while insensitive (or sensitive) to the others. This property becomes the basis to solve the isolation problem. As mentioned earlier, because UIO can decouple some unknown input signals so that it is insensitive to them, it has the potential to produce the needed structured residual signals for sensor faults isolation. One remark is that this scheme can only isolate a single or a certain group of faults. It can not isolate simultaneous faults. This is called weak sensor faults isolation.

Consider a linear system with sensor faults and disturbance as follows:

(5) $$\begin{array}{c} \begin{array}{cc} \dot{x}\left(t\right) & =Ax(t)+Bu(t)+Ed(t)\\ y(t) & =Cx\left(t\right)+f_{s}\left(t\right) \end{array} \end{array}$$

To apply the UIO for weak sensor faults isolation, we delete one by one of the rows in the *C* matrix in Equation (5) related with each sensor fault as shown in Equation (6) below:

(6) $$y^{i}\left(t\right)=C^{i}x\left(t\right)+f_{s}^{i}\left(t\right)$$

where $C^{i}\in R^{n\times \left(m-1\right)}$ is the *C* matrix without the *i*th row, $y^{i}\left(t\right)\in R^{m-1}$ is y vector without the *i*th entry, and $f_{s}^{i}\left(t\right)\in R^{m-1}$ is $f_{s}\left(t\right)$ vector without the *i*th entry.

Then, a bank of *m* UIOs for the system can be derived with the following dynamics:

(7) $$\begin{array}{c} \begin{array}{cc} \dot{z^{i}}\left(t\right) & =F^{i}z^{i}\left(t\right)+T^{i}Bu\left(t\right)+K^{i}y^{i}\left(t\right)\\ r^{i}\left(t\right) & =\left(I-C^{i}H^{i}\right)y^{i}\left(t\right)-C^{i}z^{i}\left(t\right) \end{array} \end{array}$$

with i=1,2,3,...,m; $z^{i}\left(t\right)\in R^{n}$ is the *i*th sensor fault UIO’s state vector; $r^{i}\left(t\right)\in R^{m-1}$ is the *i*th sensor fault UIO’s residual signal; and the $F^{i}$, $T^{i}$, $K^{i}$, and $H^{i}$ matrices are chosen in such a way to satisfy the following conditions:

(8) $$\begin{array}{c} \begin{array}{cc} H^{i}C^{i}E & =E\\ T^{i} & =I-H^{i}C^{i}\\ F^{i} & =T^{i}A-K_{1}^{i}C^{i}\\ K_{2}^{i} & =F^{i}H^{i}\\ K^{i} & =K_{1}^{i}+K_{2}^{i} \end{array} \end{array}$$

where $K_{1}^{i}$ is chosen to stabilize $F^{i}$.

Remark that Equation (7) shows $z^{i}\left(t\right)$ is affected by all inputs except the *i*th entry related to a specific sensor fault ($y^{i}\left(t\right)$). If a fault occurs, all the residual signals will be triggered except for the *i*th one. Hence, this bank of UIOs can be used as residual generators for weak sensor faults isolation.

## 3. Sensor Faults Isolation in Networks of Control Systems

### 3.1. Sensor Faults in Networked Control Systems

In networked control systems, besides the local control inputs, the states of each subsystem in that network are also affected by the states of its neighbouring subsystems. This is shown in Equation (9) as follows:

(9) $$\begin{array}{c} \begin{array}{cc} {\dot{x}}_{j} & =A_{j}x_{j}+B_{j}u_{j}+I_{j}i_{j}\\ y_{j} & =C_{j}x_{j} \end{array} \end{array}$$

where subscript j=1,2,3,...,N represents *j*th subsystem, $x_{j}\in R^{n}$ is the state vector, $y_{j}\in R^{m}$ is the measured output vector, $u_{j}\in R^{r}$ is the control input vector, and $i_{j}\in R^{p}$ is the interconnection input vector which represents the states from the neighbouring subsystems. $I_{j}$ is an interconnection input matrix with appropriate dimension.

By assuming static linear interconnections, the interconnection inputs $i_{j}$ can be generally written as:

(10) $$\begin{array}{c} \left.\begin{array}{cc} i(t) & =Ly(t)\\ \left[\begin{array}{c} i_{1}\left(t\right)\\ i_{2}\left(t\right)\\ i_{3}\left(t\right)\\ .\\ .\\ .\\ i_{N}\left(t\right) \end{array}\right] & =\left[\begin{array}{ccccccc} 0 & L_{12} & L_{13} & . & . & . & L_{1N}\\ L_{21} & 0 & L_{23} & . & . & . & L_{2N}\\ L_{31} & L_{32} & 0 & . & . & . & L_{3N}\\ . & . & . & . & . & . & .\\ . & . & . & . & . & . & .\\ . & . & . & . & . & . & .\\ L_{N1} & L_{N2} & L_{N3} & . & . & . & 0 \end{array}\right]\left[\begin{array}{c} y_{1}\left(t\right)\\ y_{2}\left(t\right)\\ y_{3}\left(t\right)\\ .\\ .\\ .\\ y_{N}\left(t\right) \end{array}\right] \end{array}\right. \end{array}$$

where *L* is called the interconnection matrix. This *L* matrix contains the matrices $L_{jk}$ which describes the relations between output measurements from *k*th subsystem being connected as interconnection inputs to *j*th subsystem.

For *N* connected Linear Time Invariant (LTI) subsystems subject to sensor faults and measurement noises, each of them has dynamics described by state-space equations as follows:

(11) $$\begin{array}{c} \begin{array}{cc} {\dot{x}}_{j} & =A_{j}x_{j}+B_{j}u_{j}+I_{j}\left(i_{j}+\delta i_{j}\right)\\ y_{j} & =C_{j}x_{j}+f_{\mathrm{sj}}+w_{j} \end{array} \end{array}$$

where $f_{\mathrm{sj}}\in R^{m}$ is the fault sensor vector, $w_{j}\in R^{m}$ is the measurement noises vector, and $\delta i_{j}\in R^{p}$ is the disturbance term that describes the sensor faults propagation through network such that:

(12) $$\delta i_{j}=\sum_{k=1}^{N}L_{jk}\left(f_{sk}+w_{k}\right)$$

Now, consider a general linear control algorithm where the control inputs $u_{j}$ depend on the state variable which is obtained from the output measurements $y_{j}$ so that:

(13) $$u_{j}=K_{j}y_{j}+u_{\mathrm{cj}}$$

where $K_{j}$ is the controller gain matrix and $u_{\mathrm{cj}}$ is a feed-forward term.

Hence, by substituting Equations (12) and (13) into Equation (11) and rearranging it, it can be seen that the sensor faults are now affecting the dynamics of the subsystem as follows:

(14) $$\begin{array}{c} \begin{array}{cc} {\dot{x}}_{j} & =\left[\begin{array}{c} A_{j}+B_{j}K_{j}C_{j} \end{array}\right]x_{j}+\left[\begin{array}{cc} B_{j} & I_{j} \end{array}\right]\left[\begin{array}{c} u_{\mathrm{cj}}\\ i_{j} \end{array}\right]+\left[\begin{array}{c} I_{j}\delta i_{j}+B_{j}K_{j}f_{\mathrm{sj}}+B_{j}K_{j}w_{j} \end{array}\right]\\ y_{j} & =C_{j}x_{j}+f_{\mathrm{sj}}+w_{j} \end{array} \end{array}$$

### 3.2. UIO for Sensor Faults Isolation in Networked Control Systems

In this section, we show that the UIO-based weak sensor faults isolation scheme presented in the earlier section can be applied to develop proper sensor faults isolation despite fault propagation through the network. The resulting observer can be implemented in a distributed way which means that it uses local subsystem’s measurements and the measurements of the neighbouring subsystems. The model in Equation (14) can be written in a more compact way as follows:

(15) $$\begin{array}{c} \begin{array}{cc} {\dot{x}}_{j} & ={\hat{A}}_{j}x_{j}+\hat{B_{j}}\hat{u_{j}}+\hat{E_{j}}\hat{d_{j}}\\ y_{j} & =C_{j}x_{j}+f_{\mathrm{sj}}+w_{j} \end{array} \end{array}$$

where

(16) $$\begin{array}{c} \begin{array}{cc} {\hat{A}}_{j} & =A_{j}+B_{j}KC_{j}\\ \hat{B_{j}} & =\left[\begin{array}{cc} B_{j} & I_{j} \end{array}\right]\\ \hat{E_{j}} & =\left[\begin{array}{cc} I_{j} & B_{j}K_{j} \end{array}\right]\\ \hat{d_{j}} & =\left[\begin{array}{c} \delta i_{j}\\ f_{\mathrm{sj}}+w_{j} \end{array}\right]\\ \hat{u_{j}} & =\left[\begin{array}{c} u_{\mathrm{cj}}\\ i_{j} \end{array}\right] \end{array} \end{array}$$

Remark that Equation (15) has the same form as Equation (5) such that the weak sensor faults isolation scheme can be applied directly. In addition, it is also seen that the input $\hat{u_{j}}$ depends only on the control inputs of the *j*th subsystem and the measured outputs of the neighbouring subsystems.

### 3.3. Threshold Computation for Fault Isolation

As previously explained, to perform weak sensor faults isolation, the UIOs are treated as residual generators in which each residual is insensitive to a specific fault while sensitive to the others. Consequently, because we determine the faulty sensor by observing noisy measurement signals, a suitable threshold need to be specified. By substituting Equation (6) into Equation (7), we get:

(17) $$\begin{array}{c} \begin{array}{cc} \dot{z^{i}}\left(t\right) & =F^{i}z^{i}\left(t\right)+T^{i}Bu\left(t\right)+K^{i}\left(C^{i}x\left(t\right)+f_{s}^{i}\left(t\right)+w^{i}\left(t\right)\right)\\ r^{i}\left(t\right) & =\left(I-C^{i}H^{i}\right)\left(C^{i}x\left(t\right)+f_{s}^{i}\left(t\right)+w^{i}\left(t\right)\right)-C^{i}z^{i}\left(t\right) \end{array} \end{array}$$

Thus, the sole effect of the measurement noises $\dot{z_{w}^{i}}\left(t\right)$ and $r_{w}^{i}\left(t\right)$ on the residuals are defined by the following equation:

(18) $$\begin{array}{c} \begin{array}{cc} \dot{z_{w}^{i}}\left(t\right) & =F^{i}z^{i}\left(t\right)+K^{i}w^{i}\left(t\right)\\ r_{w}^{i}\left(t\right) & =\left(I-C^{i}H^{i}\right)w^{i}\left(t\right)-C^{i}z^{i}\left(t\right) \end{array} \end{array}$$

To minimize the occurrence of false alarm situation, the highest peak gain of the system’s frequency response is chosen as threshold values ($th^{i}$) as follows:

(19) $$th^{i}=∥G_{rw}^{i}{∥}_{\infty }{∥w^{i}∥}_{\infty }$$

where $∥G_{rw}^{i}{∥}_{\infty }$ is the infinity norm of the system in Equation (18).

Hence, the weak sensor faults isolation is achieved by formulating the decision signal $\sigma_{i}$ in which the fault happened at the *i*th sensor using the relation:

(20) $$\sigma_{i}=\left\{\begin{array}{cc} 1 & \mathrm{if}\;∥r^{i}\left(t\right)∥\leq th^{i}\;\mathrm{and}\;∥r^{\hat{i}}\left(t\right)∥>th^{\hat{i}},\;\forall \hat{i}\left(t\right)\neq i\\ 0 & \mathrm{otherwise}. \end{array}\right.$$

## 4. Modelling of Controlled Robots Platoon

### 4.1. Fault Free Model of Controlled Robots Platoon

In this paper, the subsystems in networked control systems are represented by robots in a controlled robots platoon. The modelling of this controlled robots platoon is inspired by Adaptive Cruise Control (ACC) which is used for vehicle following system [32]. Several robots are assumed to move along *x* axis and each of them has a double integrator dynamic as follows:

(21) $$m_{j}{\ddot{x}}_{j}=u_{j}$$

where j=2,3,4,...,n represents the *j*th follower robot with *n* being the number of robots (j=1 is allocated to represent the leader robot), ${\ddot{x}}_{j}={\dot{v}}_{j}=a_{j}$ with $x_{j}$ is position, $v_{j}$ is velocity, $a_{j}$ is acceleration, $m_{j}$ is mass, and $u_{j}$ is the control input of the *j*th robot.

According to [30], two conditions that must be achieved in this leader-following control system are individual stability and string stability.

**Figure 1:**
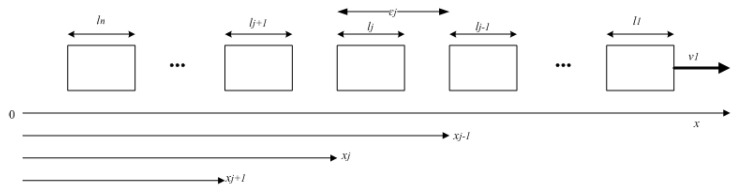
Robots platoon block diagram.

Individual stability means that the spacing error of the concerned robot should converge to zero when the preceding robot has a constant speed. The spacing error describing the distance with the preceding robot can be expressed as:

(22) $$\begin{array}{c} \begin{array}{cc} \epsilon_{j} & =x_{j}-x_{j-1}+l_{j-1}\\ e_{j} & =x_{j}-x_{j-1}+L_{j} \end{array} \end{array}$$

where $\epsilon_{j}$ is the measured inter-robot spacing, $e_{j}$ is the spacing error of the *j*th robot, and $L_{j}$ is some desired value of inter-robot spacing and includes the length of the preceding robot $l_{j-1}$. Figure 1 shows the block diagram of the concerned robots platoon.

Meanwhile, string stability means that the spacing errors on each robot should not amplify towards the end of the string ($∥e_{j}{∥}_{\infty }\leq {∥e_{j-1}∥}_{\infty }$). To satisfy both individual stability and string stability, a constant time-gap policy must used to formulate the control input for each robot. In this policy, the desired inter-robot spacing $L_{j}$ is not made constant. Instead, it varies in proportional with velocity as well as the spacing error $e_{j}$ as follows:

(23) $$\begin{array}{c} \begin{array}{cc} L_{j} & =l_{j-1}+h{\dot{x}}_{j}\\ e_{j} & =\epsilon_{j}+h{\dot{x}}_{j} \end{array} \end{array}$$

where *h* is a prescribed positive constant parameter called time-gap and $\epsilon_{j}$ is as given in the Equation (22).

A previous research [30] found that a suitable control input $u_{j}$ for this constant time-gap policy is in the form of:

(24) $$\begin{array}{c} \begin{array}{cc} u_{j}=\ddot{x_{j}} & =-\frac{1}{h}\left({\dot{\epsilon }}_{j}+\lambda e_{j}\right)\\ & =-\frac{1}{h}\left({\dot{x}}_{j}-{\dot{x}}_{j-1}+{\dot{l}}_{j-1}+\lambda x_{j}-\lambda x_{j-1}+\lambda l_{j-1}+\lambda h{\dot{x}}_{j}\right)\\ & =-\frac{\lambda }{h}x_{j}+\left(-\frac{1}{h}-\lambda \right){\dot{x}}_{j}+\frac{\lambda }{h}x_{j-1}+\frac{1}{h}{\dot{x}}_{j-1}+\left(-\frac{\lambda }{h}\right)l_{j-1} \end{array} \end{array}$$

where λ is a constant chosen such that λ>0.

For the sake of generality, Equation (24) is rewritten as:

(25) $$u_{j}=k_{1j}x_{j}+k_{2j}{\dot{x}}_{j}+k_{3j}x_{j-1}+k_{4j}{\dot{x}}_{j-1}+k_{5j}l_{j-1}$$

where $k_{1j}=-\frac{\lambda }{h}$, $k_{2j}=-\frac{1}{h}-\lambda$, $k_{3j}=\frac{\lambda }{h}$, $k_{4j}=\frac{1}{h}$, and $k_{5j}=-\frac{\lambda }{h}$.

Hence, the related state-space model for the *j*th robot is:

(26) $$\left[\begin{array}{c} {\dot{x}}_{1j}\\ {\dot{x}}_{2j}\\ {\dot{x}}_{3j} \end{array}\right]=\left[\begin{array}{ccc} 0 & 1 & 0\\ k_{1j}/m_{j} & k_{2j}/m_{j} & 0\\ 0 & 1 & 0 \end{array}\right]\left[\begin{array}{c} x_{1j}\\ x_{2j}\\ x_{3j} \end{array}\right]+\left[\begin{array}{ccc} 0 & 0 & 0\\ k_{5j}/m_{j} & k_{3j}/m_{j} & k_{4j}/m_{j}\\ 0 & 0 & -1 \end{array}\right]\left[\begin{array}{c} l_{j-1}\\ x_{j-1}\\ {\dot{x}}_{j-1} \end{array}\right]$$

where $x_{1j}=x_{j}$, $x_{2j}={\dot{x}}_{j}$, and $x_{3j}=x_{j}-x_{j-1}$.

### 4.2. Model of Controlled Robots Platoon with Sensor Faults

To implement the robots platoon’s control system, it is assumed that each robot is equipped with conventional sensors which are a GPS-based sensor, a velocity sensor, and a radar sensor. Thus, the considered measurement outputs are:

1. $y_{1j}$ is a position measurement to obtain the $x_{1j}$ state using a GPS-based sensor (S1).
2. $y_{2j}$ is a velocity measurement obtained from the same GPS-based sensor. For GPS-based velocity measurement, please refer to [33].
3. $y_{3j}$ is a velocity measurement by using the wheel-mounted velocity sensor (S2) on the robot to obtain the $x_{2j}$ state.
4. $y_{4j}$ is an inter-vehicle distance measurement to obtain the $x_{3j}$ state using a radar-based sensor (S3).

In addition, it is also assumed that each robot receives the position information from the preceding robot through wireless inter-vehicle network communication ($x_{j-1}$). The velocity from the preceding robot can also be calculated by differentiating this information ($v_{j-1}={\dot{x}}_{j-1}$).

Note that the common use of local sensors and inter-vehicle communication introduces measurements redundancy. Velocity can either be obtained from the GPS-based sensor or measured by the wheel-mounted velocity sensor. In the same manner, the inter-vehicle distance also can either be measured directly or obtained by the difference between GPS-based measurement and the position of the preceding vehicle which is received through the wireless communication network.

Besides that, the sensors are affected by additive faults and measurement noises. The measurement noises are considered high frequency and low amplitude signals. Meanwhile, sensor faults are due to malfunctioning of sensors, the measurements signal from them is expected to have a sudden or incremental change of amplitude at the time of the incident. Thus, the faults are assumed to be high magnitude signals (e.g., sensor biases) as compared to measurements noise that has a considerable influence on the dynamic behaviour of the controlled robots platoon. Hence, the output measurements can be modelled as:

(27) $$\begin{array}{c} \begin{array}{cc} y_{j} & =C_{j}x_{j}+f_{\mathrm{sj}}+w_{j}\\ C_{j} & =\left[\begin{array}{ccc} 1 & 0 & 0\\ 0 & 1 & 0\\ 0 & 1 & 0\\ 0 & 0 & 1 \end{array}\right] \end{array} \end{array}$$

where $y_{j}={\left[y_{1j}\;y_{2j}\;y_{3j}\;y_{4j}\right]}^{T}$ is the measurement vector, $x_{j}={\left[x_{1j}\;x_{2j}\;x_{3j}\right]}^{T}$ is the state vector, $f_{\mathrm{sj}}={\left[f_{s1j}\;{\dot{f}}_{s1j}\;f_{s2j}\;f_{s3j}\right]}^{T}$ is the sensor faults vector which contains position sensor fault ($f_{s1j}$), derivative of the position sensor fault (${\dot{f}}_{s1j}$), velocity sensor fault ($f_{s2j}$), distance sensor fault ($f_{s3j}$), and $w_{j}={\left[w_{1j}\;{\dot{w}}_{1j}\;w_{2j}\;w_{3j}\right]}^{T}$ is the measurement noises vector.

The received information from the network is also considered to be affected by network disturbances as follows:

(28) $$i_{j-1}=x_{j-1}+\delta i_{j-1}$$

where $i_{j-1}$ is the received position information from the preceding robot, $x_{j-1}$ is the position information from the preceding robot, and $\delta i_{j-1}$ represents the disturbance on the signal received through the network due to the possible position sensor fault of the preceding robot.

The control input for each robot in this controlled robots platoon can be calculated from the enumerated sensor measurements which are affected both by sensor faults and network disturbance. Because of the redundancy in measurements, there are several ways to compute the control signal $u_{j}$. One possible way to compute is:

(29) $$\begin{array}{c} \begin{array}{cc} u_{j} & =k_{1j}y_{1j}+k_{2j}y_{3j}+k_{3j}i_{j}+k_{4j}{\dot{i}}_{j}+k_{5j}l_{j-1}\\ u_{j} & =k_{1j}\left(x_{1j}+f_{s1j}+w_{1j}\right)+k_{2j}\left(x_{2j}+f_{s2j}+w_{2j}\right)+k_{3j}\left(x_{j-1}+\delta i_{j-1}\right)\\ & +k_{4j}\left({\dot{x}}_{j-1}+{\dot{\delta i}}_{j-1}\right)+k_{5j}l_{j-1}\\ u_{j} & =k_{1j}x_{j}+k_{2j}{\dot{x}}_{j}+k_{3j}x_{j-1}+k_{4j}{\dot{x}}_{j-1}+k_{5j}l_{j-1}+D_{j} \end{array} \end{array}$$

where $D_{j}$ represents an unknown disturbance term which sums up the additive faults and the noises terms that arrive through the networks or from the local faulty sensors as follows:

(30) $$D_{j}=k_{1j}f_{s1j}+k_{1j}w_{1j}+k_{2j}f_{s2j}+k_{2j}w_{2j}+k_{3j}\delta i_{j-1}+k_{4j}{\dot{\delta i}}_{j-1}$$

If we implement the control input using measurements from other sensors, $u_{j}$ will have the same form as in Equation (29) and only $D_{j}$ will change.

Thus, the state-space equation with unknown disturbance term for the *j*th robot in a controlled robots platoon is:

(31) $$\begin{array}{c} \begin{array}{cc} \left[\begin{array}{c} {\dot{x}}_{1j}\\ {\dot{x}}_{2j}\\ {\dot{x}}_{3j} \end{array}\right] & =\left[\begin{array}{ccc} 0 & 1 & 0\\ k_{1j}/m_{j} & k_{2j}/m_{j} & 0\\ 0 & 1 & 0 \end{array}\right]\left[\begin{array}{c} x_{1j}\\ x_{2j}\\ x_{3j} \end{array}\right]+\left[\begin{array}{ccc} 0 & 0 & 0\\ k_{5j}/m_{j} & k_{3j}/m_{j} & k_{4j}/m_{j}\\ 0 & 0 & -1 \end{array}\right]\left[\begin{array}{c} l_{j-1}\\ x_{j-1}\\ {\dot{x}}_{j-1} \end{array}\right]\\ & +\left[\begin{array}{cccc} 0 & 0 & 0 & 0\\ k_{3j}/m_{j} & k_{4j}/m_{j} & k_{1j}/m_{j} & k_{2j}/m_{j}\\ 0 & 0 & 0 & 0 \end{array}\right]\left[\begin{array}{c} \delta i_{j-1}\\ {\dot{\delta i}}_{j-1}\\ f_{s1j}+w_{1j}\\ f_{s2j}+w_{2j} \end{array}\right]\\ \left[\begin{array}{c} y_{1j}\\ y_{2j}\\ y_{3j}\\ y_{4j} \end{array}\right] & =\left[\begin{array}{ccc} 1 & 0 & 0\\ 0 & 1 & 0\\ 0 & 1 & 0\\ 0 & 0 & 1 \end{array}\right]\left[\begin{array}{c} x_{1j}\\ x_{2j}\\ x_{3j} \end{array}\right]+\left[\begin{array}{c} f_{s1j}\\ {\dot{f}}_{s1j}\\ f_{s2j}\\ f_{s3j} \end{array}\right]+\left[\begin{array}{c} w_{1j}\\ {\dot{w}}_{1j}\\ w_{2j}\\ w_{3j} \end{array}\right] \end{array} \end{array}$$

### 4.3. Special Case: The Leader Robot

It is considered that the leader (first) robot moves with a prescribed controlled speed and it does not receive information from the other robots. In a similar manner, this leader is assumed to move along *x* axis and has a double integrator dynamic as follows:

(32) $$m_{1}{\ddot{x}}_{1}=u_{1}$$

where ${\ddot{x}}_{1}={\dot{v}}_{1}=a_{1}$ with $x_{1}$ is leader’s position, $v_{1}$ is leader’s velocity, $a_{1}$ is leader’s acceleration, $m_{1}$ is leader’s mass, and $u_{1}$ is the control input for this leader robot.

The needed control input for leader is calculated only from its sensor measurements without being affected by the states of the other robots. A proportional integrator (PI) velocity controller is assumed as it is shown in the Equation (33):

(33) $$\begin{array}{c} \begin{array}{cc} u_{1} & =k_{i}\int_{0}^{t}\left(v_{ref}-v_{1}\right)\;d\tau +k_{p}\left(v_{ref}-v_{1}\right)\\ u_{1} & =-k_{i}x_{1}-k_{p}v_{1}+u_{c}\\ u_{c} & =k_{i}\int_{0}^{t}v_{ref}\;d\tau +k_{p}v_{ref} \end{array} \end{array}$$

where $k_{i}$ is the integral control gain, $k_{p}$ is the proportional control gain, $u_{c}$ is the feed-forward control term, and $v_{ref}$ is a predefined speed set-point for the leader robot.

The related state-space model of the leader is as seen in the Equation (34).

(34) $$\left[\begin{array}{c} {\dot{x}}_{11}\\ {\dot{x}}_{21} \end{array}\right]=\left[\begin{array}{cc} 0 & 1\\ -k_{i}/m_{1} & -k_{p}/m_{1} \end{array}\right]\left[\begin{array}{c} x_{11}\\ x_{21} \end{array}\right]+\left[\begin{array}{c} 0\\ 1/m_{1} \end{array}\right]u_{c}$$

where $x_{11}=x_{1}$ and $x_{21}={\dot{x}}_{1}=v_{1}$.

### 4.4. Model of the Leader Robot with Sensor Faults

The assumed measurement outputs for this leader robot are:

1. $y_{11}$ is a GPS-based position measurement to obtain the $x_{11}$ state.
2. $y_{21}$ is a velocity measurement based on the same GPS sensor.
3. $y_{31}$ is a velocity measurement by using the wheel-mounted velocity sensor on the robot to obtain the $x_{21}$ state.

The control input of the leader only depends on it’s own sensor measurements. The unknown inputs, which act as disturbance for the faulty model of the leader robot, come from this leader’s sensor fault. With this additive sensor fault, the leader’s measurements become as shown in the Equation (35):

(35) $$\begin{array}{c} \begin{array}{cc} y_{1} & =C_{1}x_{1}+f_{s1}+w_{1}\\ C_{1} & =\left[\begin{array}{cc} 1 & 0\\ 0 & 1\\ 0 & 1 \end{array}\right] \end{array} \end{array}$$

where $y_{1}={\left[y_{11}\;y_{21}\;y_{31}\right]}^{T}$ is the leader’s measurement vector, $x_{1}={\left[x_{11}\;x_{21}\right]}^{T}$ is the leader’s state vector, $f_{s1}={\left[f_{s11}\;{\dot{f}}_{s11}\;f_{s21}\right]}^{T}$ is the leader’s sensor faults vector which contains position sensor fault ($f_{s11}$), derivative of the position sensor fault (${\dot{f}}_{s11}$), velocity sensor fault ($f_{s21}$), and $w_{1}={\left[w_{11}\;{\dot{w}}_{11}\;w_{21}\right]}^{T}$ is the leader’s measurement noises vector.

By taking into consideration the sensor faults, one possible way to compute the control input of the leader is as shown in the Equation (36):

(36) $$\begin{array}{c} \begin{array}{cc} u_{1} & =-k_{i}y_{11}-k_{p}y_{31}+u_{c}\\ u_{1} & =-k_{i}\left(x_{11}+f_{s11}+w_{11}\right)-k_{p}\left(x_{21}+f_{s21}+w_{21}\right)+u_{c}\\ u_{1} & =-k_{i}x_{11}-k_{p}x_{21}+u_{c}+D_{1} \end{array} \end{array}$$

where $D_{1}$ contains the additive fault-induced disturbance and noise terms as follows:

(37) $$D_{1}=-k_{i}f_{s11}-k_{i}w_{11}-k_{p}f_{s21}-k_{p}w_{21}$$

Thus, the state-space equation with sensor faults for the leader robot in a controlled robots platoon is as seen in Equation (38):

(38) $$\begin{array}{c} \begin{array}{cc} \left[\begin{array}{c} {\dot{x}}_{11}\\ {\dot{x}}_{21} \end{array}\right] & =\left[\begin{array}{cc} 0 & 1\\ -k_{i}/m_{1} & -k_{p}/m_{1} \end{array}\right]\left[\begin{array}{c} x_{11}\\ x_{21} \end{array}\right]+\left[\begin{array}{c} 0\\ 1/m_{1} \end{array}\right]u_{c}+\left[\begin{array}{cc} 0 & 0\\ -k_{i}/m_{1} & -k_{p}/m_{1} \end{array}\right]\left[\begin{array}{c} f_{s11}+w_{11}\\ f_{s21}+w_{21} \end{array}\right]\\ \left[\begin{array}{c} y_{11}\\ y_{21}\\ y_{31} \end{array}\right] & =\left[\begin{array}{cc} 1 & 0\\ 0 & 1\\ 0 & 1 \end{array}\right]\left[\begin{array}{c} x_{11}\\ x_{21} \end{array}\right]+\left[\begin{array}{c} f_{s11}\\ {\dot{f}}_{s11}\\ f_{s21} \end{array}\right]+\left[\begin{array}{c} w_{11}\\ {\dot{w}}_{11}\\ w_{21} \end{array}\right] \end{array} \end{array}$$

## 5. Sensor Faults Isolation in Controlled Robots Platoon

### 5.1. Sensor Faults Isolation in the Follower Robots

In the case of controlled robots platoon, the network disturbance yields from the position sensor fault measurement of the preceding robot which is transmitted to the robot behind it. For the *j*th robot, we can treat both the network disturbance from the preceding (j−1)th robot and the effect of its own sensor faults on control as unknown input part $d_{j}$. By comparing Equation (15) with Equation (31), we can write:

(39) $$\begin{array}{c} \begin{array}{cc} {\hat{A}}_{j} & =\left[\begin{array}{ccc} 0 & 1 & 0\\ k_{1j}/m_{j} & k_{2j}/m_{j} & 0\\ 0 & 1 & 0 \end{array}\right]\\ {\hat{B}}_{j} & =\left[\begin{array}{ccc} 0 & 0 & 0\\ k_{5j}/m_{j} & k_{3j}/m_{j} & k_{4j}/m_{j}\\ 0 & 0 & -1 \end{array}\right]\\ \hat{E_{j}} & =\left[\begin{array}{cccc} 0 & 0 & 0 & 0\\ k_{3j}/m_{j} & k_{4j}/m_{j} & k_{1j}/m_{j} & k_{2j}/m_{j}\\ 0 & 0 & 0 & 0 \end{array}\right]\\ x_{j} & =\left[\begin{array}{c} x_{1j}\\ x_{2j}\\ x_{3j} \end{array}\right]\\ \hat{u_{j}} & =\left[\begin{array}{c} l_{j-1}\\ x_{j-1}\\ {\dot{x}}_{j-1} \end{array}\right]\\ \hat{d_{j}} & =\left[\begin{array}{c} \delta i_{j-1}\\ {\dot{\delta i}}_{j-1}\\ f_{s1j}+w_{1j}\\ f_{s2j}+w_{2j} \end{array}\right] \end{array} \end{array}$$

The output matrix $C_{j}$ is given by Equation (27). The $C_{j}^{1}$, $C_{j}^{2}$, and $C_{j}^{3}$ matrices, which correspond to the design of UIO for weak sensor faults isolation, are obtained in the same manner as in Equation (6) so that we get:

(40) $$\begin{array}{c} \begin{array}{cc} C_{j}^{1} & =\left[\begin{array}{ccc} 0 & 1 & 0\\ 0 & 0 & 1 \end{array}\right]\\ C_{j}^{2} & =\left[\begin{array}{ccc} 1 & 0 & 0\\ 0 & 1 & 0\\ 0 & 0 & 1 \end{array}\right]\\ C_{j}^{3} & =\left[\begin{array}{ccc} 1 & 0 & 0\\ 0 & 1 & 0\\ 0 & 1 & 0 \end{array}\right] \end{array} \end{array}$$

One remark for UIO which is designed to be insensitive to a fault in S1 is that both the 1st and 2nd row of the *C* matrix is deleted to form $C_{j}^{1}$ because $y_{2j}$ represents velocity which is obtained from the same sensor to obtain $y_{1j}$. Thus, GPS-based velocity measurement is not treated as an extra sensor that needs faults isolation.

**Figure 2:**
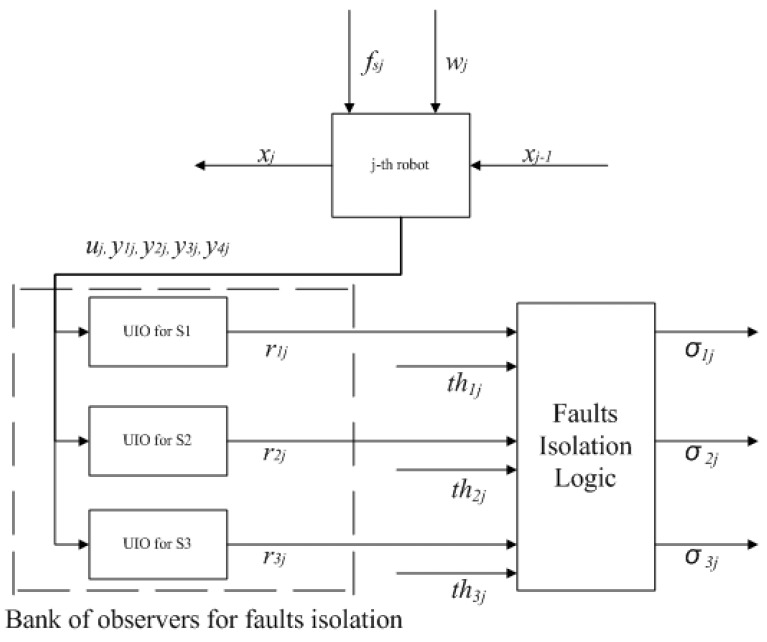
Local UIOs for sensor faults isolation on each robot.

By using each of those three new $C_{j}$ matrices, three UIOs for each sensor faults isolation can be designed for each robot according to Equation (7) to produce residual signals $r_{1j}$, $r_{2j}$, and $r_{3j}$ which are insensitive to a fault in S1, S2, and S3, respectively. Afterwards, these residual signals are compared to each threshold value $th^{i}$ computed from Equation (19). When a fault occurs, the decision signal $\sigma_{i}$ will rise based on that comparison results as shown in Equation (20). For example, if the residual signals from S1 ($r_{1j}$) and S3 ($r_{3j}$) are higher than the threshold values while a residual signal from S2 ($r_{2j}$) is lower, then decision signal for S2 ($\sigma_{2}$) will rise from zero to one indicating that a fault is happening in S2. The block diagram of this bank of UIOs along with its isolation logic is shown in Figure 2.

### 5.2. Sensor Faults Isolation in the Leader Robot

Sensor faults isolation for the leader robot has a rather different scheme because its states are not affected by the states of the other robots. One more thing to be noted is that it only has redundancy in velocity measurement which comes from both the GPS-based sensor measurement (S1) and the wheel-mounted velocity sensor measurement (S2).

Because the leader robot has redundancy in the velocity measurement, we can directly use the method as in the case of the follower robots to design the UIO which is insensitive to fault in S2. By comparing Equation (15) with Equation (38), we use these matrices in constructing the UIO which produces $r_{21}$ as follows:

(41) $$\begin{array}{c} \begin{array}{cc} {\hat{A}}_{1}^{2} & =\left[\begin{array}{cc} 0 & 1\\ -k_{i}/m_{1} & -k_{p}/m_{1} \end{array}\right]\\ {\hat{B}}_{1}^{2} & =\left[\begin{array}{c} 0\\ 1/m_{1} \end{array}\right]\\ C_{1}^{2} & =\left[\begin{array}{cc} 1 & 0\\ 0 & 1 \end{array}\right]\\ {\hat{E}}_{1}^{2} & =\left[\begin{array}{cc} 0 & 0\\ -k_{i}/m_{1} & -k_{p}/m_{1} \end{array}\right]\\ x_{1}^{2} & =\left[\begin{array}{c} x_{11}\\ x_{21} \end{array}\right]\\ \hat{u_{1}^{2}}=u_{c} & =k_{i}\int_{0}^{t}v_{ref}\;d\tau +k_{p}v_{ref}\\ \hat{d_{1}^{2}} & =\left[\begin{array}{c} f_{s11}+w_{11}\\ f_{s21}+w_{21} \end{array}\right] \end{array} \end{array}$$

As the position measurement of the leader does not have any redundancy, we refer to Equation (36) to design a residual generator for fault isolation in S1 by neglecting the disturbance parts $E_{1}^{1}$ and $D_{1}$ so that the produced residual signal will respond to any sensor bias fault in S2. We can do that because we perform weak sensor faults isolation that assumes no simultaneous fault. Thus, we get:

(42) $${\dot{x}}_{21}=-\frac{k_{p}}{m_{1}}x_{21}-\frac{k_{i}}{m_{1}}x_{11}+\frac{1}{m_{1}}u_{c}$$

After that, by comparing the equation above with Equation (15), the matrices to design the UIO to produce residual signal $r_{11}$ are:

(43) $$\begin{array}{c} \begin{array}{cc} {\hat{A}}_{1}^{1} & =-k_{p}/m_{1}\\ {\hat{B}}_{1}^{1} & =\left[\begin{array}{cc} -k_{i}/m_{1} & 1/m_{1} \end{array}\right]\\ C_{1}^{1} & =1\\ {\hat{E}}_{1}^{1} & =0\\ x_{1}^{1} & =x_{21}\\ \hat{u_{1}^{1}} & =\left[\begin{array}{c} x_{11}\\ u_{c} \end{array}\right]\\ u_{c} & =k_{i}\int_{0}^{t}v_{ref}\;d\tau +k_{p}v_{ref} \end{array} \end{array}$$

## 6. Results and Discussion

### 6.1. Simulation Method

To verify the behaviour of the proposed model and the performance of the proposed weak sensor faults isolation method, we performed simulations using the MATLAB programming environment. In these simulations, the controlled robots platoon consists of five robots where one robot acts as the leader while the remaining four robots act as the followers. Each of them transmits its GPS-based position measurement to the robot behind it.

Firstly, the M-file feature in MATLAB is used to initialize the state-space parameters of both the leader and followers robots, to check the fulfilment of the UIO’s existence requirement, and, if it exists, to compute the required parameters of the UIO’s matrices for weak sensor faults isolation purpose. In this simulation, the parameters for the controlled leader robot in both Equations (41) and (43) are: $k_{i}=0.05$, $k_{p}=2$, and $m_{1}=1$ where the values of $k_{i}$ and $k_{p}$ are the PI controller gain values for leader’s velocity which is determined to get fast settling time with small overshoot.

Meanwhile, the parameters for the remaining four of the follower robots in Equation (39) are obtained from Equation (25) in which $m_{j}=1$, $l_{j-1}=3$, h=2, and λ=20 refers to the adaptive cruise control examples in [30]. It yields that: $k_{1j}=-10$, $k_{2j}=-20.5$, $k_{3j}=10$, $k_{4j}=0.5$, and $k_{5j}=-10$.

Because each robot sends its GPS-based position measurement (S1) to the robot behind it, the interconnection matrix *L* in the Equation (10) is:

$$L=\left[\begin{array}{cccccc} 0 & 0 & 0 & 0 & 0 & 0\\ 1 & 0 & 0 & 0 & 0 & 0\\ 0 & 1 & 0 & 0 & 0 & 0\\ 0 & 0 & 1 & 0 & 0 & 0\\ 0 & 0 & 0 & 1 & 0 & 0 \end{array}\right]$$

By using the new *C* matrices as in Equations (40), (41) and (43), the rank and detectability conditions necessary to UIO existence in Equation (4) are checked and it turns out that they are fulfilled.

Subsequently, the UIOs $H_{j}^{i}$, $T_{j}^{i}$, $F_{j}^{i}$, and $K_{j}^{i}$ matrices are computed to design residual generators where *i* represents *i*th sensor and *j* represents *j*th robot. Hence, the UIO-based residual generators for each sensor in each robot as shown in Figure 2 are implemented based on those matrices. The observer gains were determined using pole placement to satisfy all the equations in Equation (8) with poles: at −15 for the leader’s sensor S1; at −15 and −10 for the leader’s sensor S2; at −15 and −10 for both the follower’s sensor S1 and sensor S3; and poles at −15, −10, and −5 for the follower’s sensor S2.

After that, Equations (18) and (19) are used to calculate the residuals threshold by considering measurement noises such that $∥w^{i}{∥}_{\infty }=0.35$ in the simulation. The infinity norm gain values of the residual generators are 1 for the followers and 2.6 for the leader. By observing the produced residual signals from the UIOs and comparing them with the threshold values, weak sensor faults isolation are done using the detection/isolation logic shown in Equation (20). Based on that relation, assuming a single sensor fault happens in a robot, the decision signal related to the malfunctioning sensor will arise.

Lastly, the Simulink feature in MATLAB is used to build and simulate the controlled robots platoon model with sensor faults and fault diagnostics. The computation results of UIOs are passed to the Simulink model which represents the residual generators.

### 6.2. Simulation Results

**Figure 3:**
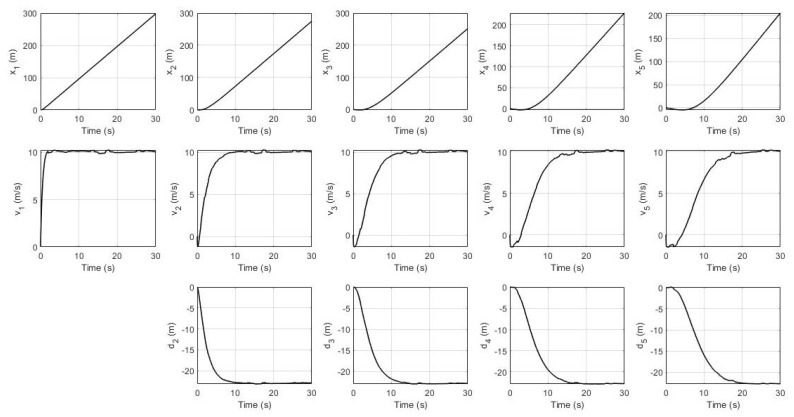
System’s response without fault.

**Figure 4:**
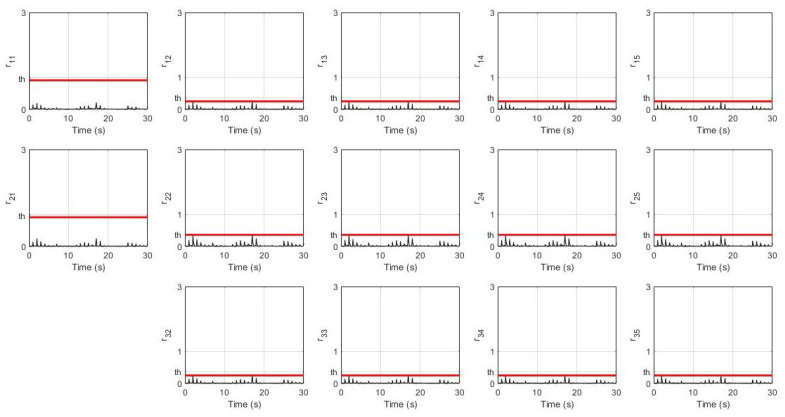
Residual signals without fault.

In this simulation, the leader’s desired velocity was set to 10 m/s. For the non-faulty case, the system’s response for every robot (position, velocity, and distance) and their residual signals (residual signal for S1, residual signal for S2, and residual signal for S3) are shown in Figure 3 and Figure 4 respectively. It can be seen that all of them achieve the desired velocity of 10 m/s and have a distance of 22.4 m between each robot with a slower settling time consecutively. Furthermore, all residual signals show very small amplitudes below the threshold indicating that no false alarms are produced.

Meanwhile, for the faulty case, we simulate four different scenarios. In these scenarios, all of the sensor faults are simulated as sensor bias errors using a step function where the magnitude is increased from 0 to 10 at the 10th second.

**Figure 5:**
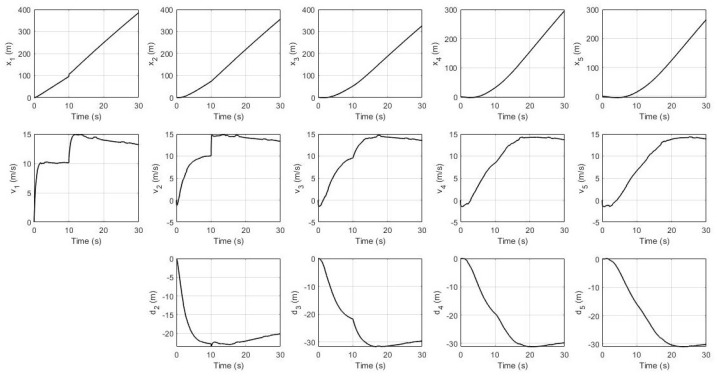
System’s response with sensor S1 fault in the 1st robot.

**Figure 6:**
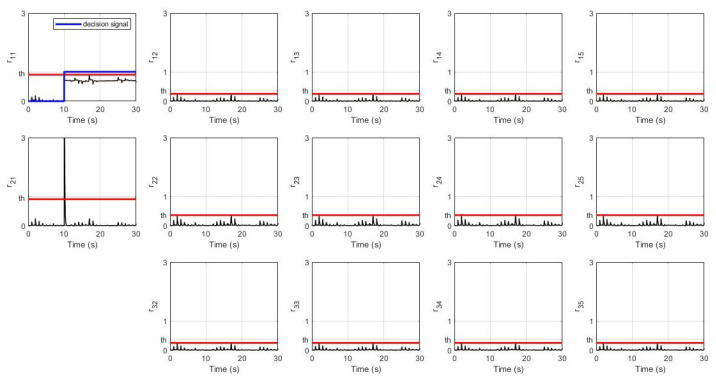
Residual signals with sensor S1 fault in the 1st robot.

In the first scenario, the fault is happening at sensor S1 in the 1st robot (leader) by which the system’s response and UIO’s residual signals are shown in Figure 5 and Figure 6 respectively.

**Figure 7:**
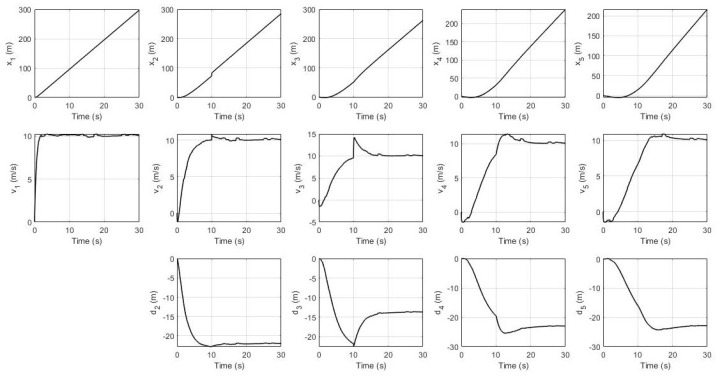
System’s response with sensor S1 fault in the 2nd robot.

**Figure 8:**
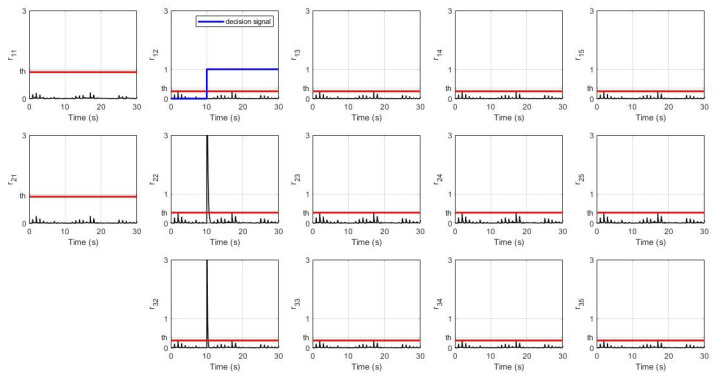
Residual signals with sensor S1 fault in the 2nd robot.

In the second scenario, the fault is happening at sensor S1 in the 2nd robot by which the system’s response and UIO’s residual signals are shown in Figure 7 and Figure 8 respectively.

**Figure 9:**
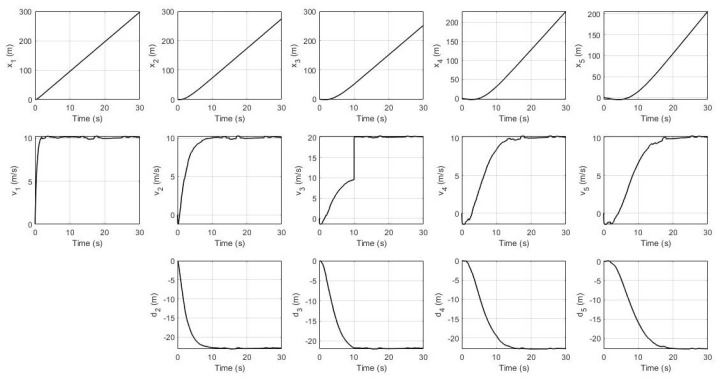
System’s response with sensor S2 fault in the 3rd robot.

**Figure 10:**
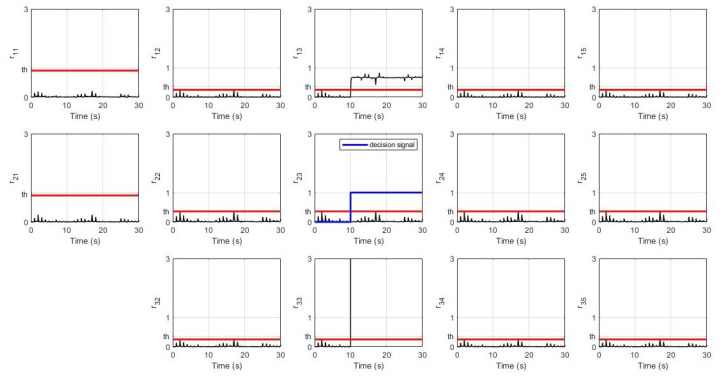
Residual signals with sensor S2 fault in the 3rd robot.

In the third scenario, the fault is happening at sensor S2 in the 3rd robot by which the system’s response and UIO’s residual signals are shown in Figure 9 and Figure 10 respectively.

**Figure 11:**
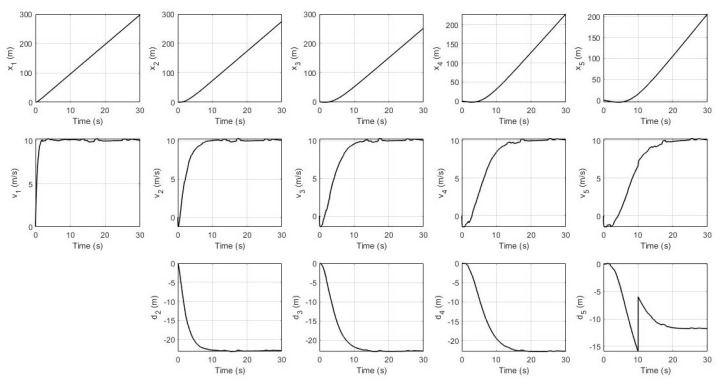
System’s response with sensor S3 fault in the 5th robot.

**Figure 12:**
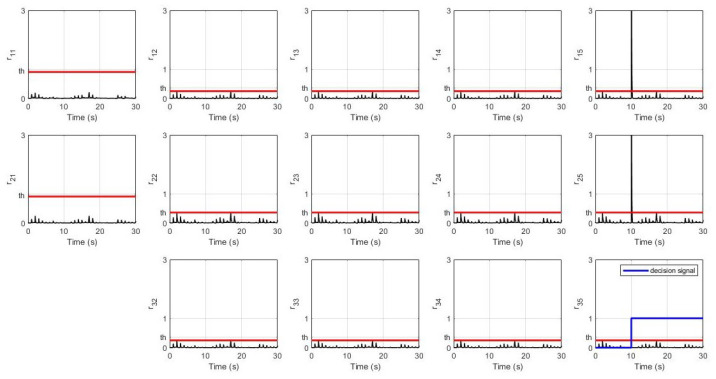
Residual signals with sensor S3 fault in the 5th robot.

Finally, in the fourth scenario, the fault is happening at sensor S3 in the 5th robot by which the system’s response and UIO’s residual signals are shown in Figure 11 and Figure 12 respectively.

For the first scenario, Figure 5 shows that a fault in S1 in the 1st robot is affecting all the states (position, velocity, and distance) of the 2nd, 3rd, 4th, and 5th robots. This is happening because, as mentioned earlier, the transmitted information in these networked control systems comes from this GPS-based position measurement (S1). Besides that, Figure 6 shows a significant change of amplitude in the residual signals produced by the UIO for sensor S2 while the other residual for sensor S1 is below the threshold value. Thus, based on the previous relation shown in the Equation (20), a decision signal for sensor S1 arise indicating that a fault occurred in this sensor at the 10th second.

For the second scenario, Figure 7 shows a similar situation as in the first scenario where a fault in S1 in the 2nd robot is affecting all the states of the 3rd, 4th, and 5th robots. In terms of sensor faults isolation, Figure 8 shows a significant change of amplitude in the residual signals for both sensor S2 and sensor S3 exceeding the threshold value while residual for sensor S1 is not. Hence, the decision signal for S1 arises revealing an occurrence of a fault in this sensor of the 2nd robot at the 10th second.

For the third scenario, Figure 9 shows that fault in S2 in the 3rd robot does not directly affect the states of the 4th and 5th robots because the measurement of this sensor (S2) is not the information that is transmitted to the robot behind it. In addition, Figure 10 shows a change of amplitude exceeding the threshold in both the residual signals from this robot’s UIO for sensor S1 and sensor S3 triggering a decision signal to indicate that sensor S2 is faulty at the 10th second.

Lastly, for the fourth scenario, Figure 11 shows that fault in S3 in the 5th robot does not directly affect any other robots at all because this 5th robot is the last in these networked control systems. Furthermore, Figure 12 shows a significant change of amplitude in this last robot’s UIO residual signal for both sensor S1 and sensor S2 triggering a decision signal to indicate that sensor S3 is faulty at the 10th second.

### 6.3. Discussion

The simulation results presented in the previous section show that the proposed faults isolation method ensures fast response time, the interval between the rise of the fault signal and the rise of the decision signal is less than 5 ms in each case. Moreover, those decision signals are always correct and accurate in determining where the fault is happening. In the case of the robot platoons, the UIO existence conditions are fulfilled.

Comparing with other similar works, this UIO-based approach shows its popularity as a favourite choice in the field of fault detection and isolation in the multi-agents system. Liu et al. implemented multiple UIOs in each agent for fault detection and isolation in the multi-agents system that is robust to disturbance [27]. While their method prioritizes the detection of which agents are failed, our approach is more refined in terms of it can detect a specific sensor fault in each agent. Other works by Shames et al. also used UIO to detect a faulty agent in a power network [28]. Yet, they also did not aim to detect a specific sensor fault in an agent.

The research object of Zhang et al. is the most similar to ours compared with the others. They derived a global augmented system model included with actuator and sensor faults and then used it to design a global UIO able to undertake faults estimation processes [29]. Their proposed faults isolation logic was a centralized one. The used case study was a simulation of five aircraft in a leader-follower network scheme. Even though we are lacking in actuator faults feature compared to them, our approach of local distributed UIOs is more scalable.

## 7. Conclusions

The extension of model-based sensor fault isolation schemes to networked control systems is essential to deal with fault effect propagation through the interconnections. In this research, we proposed a networked control system model that can be used to describe the expected behaviour of the robotics platoon with sensor faults and their propagation through the network. Distributed Unknown Input Observers (UIO) for weak sensor fault isolation in each robot are also designed using this model as the basis. In designing these UIOs, the fault propagation is treated as the unknown input part. We have also introduced a threshold computation method for UIO-based sensor fault isolation in the presence of measurement noises.

The verification and validation are done in MATLAB using a case study where five subsystems (robots) are connected with a communication-based connection. In these five robots, one robot acts as the leader which has two sensors while the remaining four robots act as the followers by which each of them has three sensors. The simulation results show that the weak sensor faults isolation in the robotics platoon is successfully achieved by observing the produced residual signals from the UIOs in each robot.

Some limitations of the UIO-based faults isolation approach in networked control systems are related to the assumptions, see Equation (4), that the subsystem models have to be satisfied to decouple the propagation of the faults through the network. Besides that, the detection and isolation logic depends on a threshold value determined by the maximum value of measurements noise. Thus, if the measurements noise has excessive values, the threshold value will also be high so that low magnitude fault can not be detected.

In many communication networks, the delay can not be neglected. According to the previous works, if the delay is small enough (under 250 ms), then the model of the system with small delay can be approximated with a model without delay [34]. Hence, if the delay in the communication network between the subsystems is small, we can implement our fault isolation approach based on the approximated model.

As the proposed model for faults isolation has low complexity, this approach has a great potential to be implemented on embedded systems. The bank of UIOs that perform the faults isolation can be discretized using standard methods. Furthermore, the approach utilizes only conventional sensors which are available on most mobile robots.

Possible future works include exploring this approach to perform actuator fault isolation in networked control systems. Another possible extension is to take into consideration the communication time delay in the form of distributed delay among the subsystems.

## Data Availability

Not applicable.
